# Who does not participate in a follow-up postal study? a survey of infertile couples treated by in vitro fertilization

**DOI:** 10.1186/1471-2288-12-104

**Published:** 2012-07-23

**Authors:** Penelope Troude, Estelle Bailly, Juliette Guibert, Jean Bouyer, Elise de La Rochebrochard

**Affiliations:** 1 INED, 133 boulevard Davout, 75940, Paris Cedex 20, France; 2 INSERM, Center for Research in Epidemiology and Population Health, CESP U1018, 94276, Le Kremlin-Bicêtre, France; 3 Univ Paris Diderot, Sorbonne Paris Cité, Service de Santé Publique et Economie de la Santé, 75475, Paris, France; 4 Laboratoire de Procréation Médicalement Assistée, Institut Mutualiste de Montsouris, 75014, Paris, France; 5 Univ Paris-Sud, UMRS 1018, 94276, Le Kremlin-Bicêtre, France

**Keywords:** Infertility, IVF, Postal survey, Response rate

## Abstract

**Background:**

A good response rate has been considered as a proof of a study’s quality. Decreasing participation and its potential impact on the internal validity of the study are of growing interest. Our objective was to assess factors associated with contact and response to a postal survey in a epidemiological study of the long-term outcome of IVF couples.

**Methods:**

The DAIFI study is a retrospective cohort including 6,507 couples who began an IVF program in 2000-2002 in one of the eight participating French IVF centers. Medical data on all 6,507 couples were obtained from IVF center databases, and information on long-term outcome was available only for participants in the postal survey (*n* = 2,321). Logistic regressions were used to assess firstly factors associated with contact and secondly factors associated with response to the postal questionnaire among contacted couples.

**Results:**

Sixty-two percent of the 6,507 couples were contacted and 58% of these responded to the postal questionnaire. Contacted couples were more likely to have had a child during IVF treatment than non-contactable couples, and the same was true of respondents compared with non-respondents. Demographic and medical characteristics were both associated with probability of contact and probability of response. After adjustment, having a live birth during IVF treatment remained associated with both probabilities, and more strongly with probability of response. Having a child during IVF treatment was a major factor impacting on participation rate.

**Conclusions:**

Non-response as well as non-contact were linked to the outcome of interest, i.e. long-term parenthood success of infertile couples. Our study illustrates that an a priori hypothesis may be too simplistic and may underestimate potential bias. In the context of growing use of analytical methods that take attrition into account (such as multiple imputation), we need to better understand the mechanisms that underlie attrition in order to choose the most appropriate method.

## Background

Participation rates in cohort studies have decreased during the last two decades [1]. A good response rate has been considered as a proof of a study’s quality [2]. Therefore, decreasing participation and its potential impact on the internal validity of studies are of growing interest [3-5]. Decreasing participation raises the question of “how much is too much?”, but above all, the question of “who do you lose?” [6-8].

In a substantial number of published studies, information about participation rates is not given or is incomplete, especially in cohort studies [9]. This underreporting may be in part linked to the epidemiological tendency to consider low participation rate as a sign of inferior quality [1]. Moreover, participation rates may be overestimated, since authors may define participation without taking into account all the different steps along the path of data collection [1,9]. In studies that did report participation rates, loss to follow-up was sometimes surprisingly high: the French COCON study on women’s contraception practices reported a loss of one-third of its members between the first and third waves, a period of 2 years [10]. In the UK Millennium Cohort Study, 20% of parents who participated in the first sweep did not respond to the second sweep two years later [11,12]. Participation rates lower than 50% have been reported in various follow-up studies such as the Danish National Birth Cohort [13,14], the Australian 45 and Up Study [15,16], the Australian Longitudinal Study on Women’s Health [17] and the French GAZEL cohort [18,19].

Participation rates may even be lower in specific populations such as infertile couples receiving or having received medical treatment, as they could be reluctant to participate after the end of their treatment in a study that reminds them of a physically and psychologically exhausting experience [20-23]. Because of these high non-participation rates, selection bias could be a serious issue in such studies. Research on factors associated with non-participation is quite rare because very often there is no information at all on those who did not participate [1,5,8,24]. However, if we are to use appropriate analytical methods that take non-participation into account, we need to understand its underlying mechanisms.

A recent large French retrospective cohort study was conducted by postal questionnaire among couples who had received in vitro fertilization (IVF) treatment. Information on all cohort members was collected in the IVF centers, allowing comparison of participants and non-participants. The main outcome of interest was parenthood project achievement after treatment in the inclusion IVF center (following further medical treatment elsewhere, spontaneous birth or adoption of a child). Achievement of the parenthood project may be associated with factors such as age, number of embryos obtained during IVF treatment or occurrence of a birth following medical treatment in the inclusion center. Comparison of these factors between participants and non-participants would enable us to discuss possible selection bias. We hypothesized that demographic, but not medical, characteristics may differ between contacted and non-contactable couples, whereas both may differ between respondents and non-respondents. Our objective was to assess factors associated with contact and response in an epidemiological study on the long-term outcome of couples after IVF treatment.

## Methods

### Population

This study is based on the DAIFI study (Devenir Après Initiation d’un programme de FIV, outcome after IVF initiation), a retrospective cohort exhaustively including all couples who began an IVF program between 2000 and 2002 (*n* = 6,507) in one of the eight participating French IVF centers (the centers at Besançon University Hospital, Cochin Hospital, Caen, Marseille, Sèvres, Bois-Guillaume, Clermont-Ferrand and Montsouris). Initiation of an IVF program was defined as the first oocyte retrieval carried out at the center, regardless of whether the patient had had previous IVF treatment elsewhere.

The study received approval from the French Data Protection Authority in September 2005 (authorization number 05-1334).

### Data collection

Data collection was based on IVF center medical files and on a postal questionnaire filled in by patients.

Medical data on all eligible couples (*n* = 6,507) were obtained from the IVF centers. These data included sterility assessments for the couple (age, origin, type and duration of infertility), the number of IVF attempts in the center, information on these attempts (number of oocytes retrieved, number of embryos obtained, number of embryos transferred, pregnancy) and on the outcome of any ensuing pregnancies.

Data on the couples’ long-term outcome were collected via questionnaires filled in by the patients in 2008-2010. These data included sociodemographic information and the path followed by the couple before, during and after treatment in the inclusion center.

### Analysis

Contact rate was defined as the number of couples contacted among the total number of included couples, response rate as the number of respondents among the contacted couples, and participation rate as the number of respondents among the total number of included couples. As reasons for non-contact and for non-response were not necessarily the same, a two-step analysis strategy was conducted.

Firstly, contacted couples were compared with non-contactable couples. Secondly, among contacted couples, respondents to the postal survey were compared with non-respondents. Univariate and multivariate logistic regressions were conducted to assess associated factors: woman’s age (< 30, 30-34, 35-39, ≥ 40 years), year of the first attempt in the inclusion center (2000, 2001, 2002), inclusion center, origin of infertility (female or male factor, mixed, unexplained), the total number of embryos obtained at the first attempt (0-1, 2-5, > 5), number of attempts in the inclusion center (1, 2-4, > 4) and outcome of treatment in the center (live birth or not).

Statistical analyses were performed using STATA/SE 10.0 (Stata Press, College Station, TX, USA).

## Results

**Figure 1:**
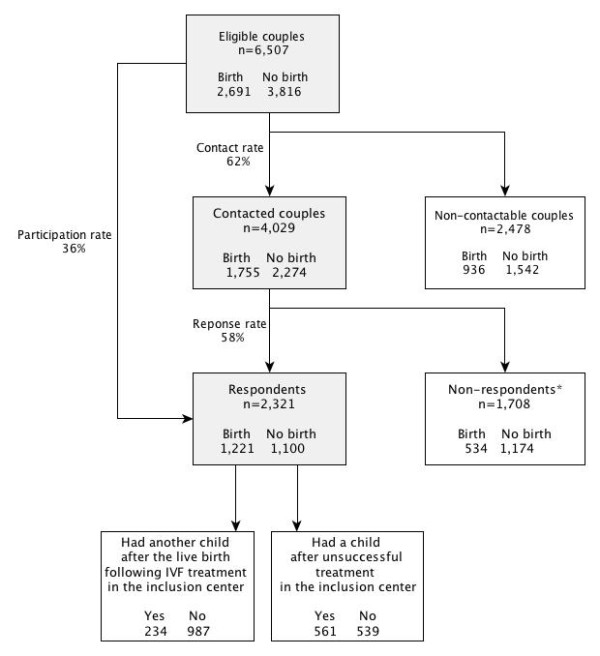
**Flow chart.**“Birth” indicates a live birth following IVF treatment in the inclusion center. “Having another child after live birth following IVF treatment in the inclusion center” or “having a child after unsuccessful treatment in the inclusion center” included: spontaneous live birth, live birth following another treatment, and adoption. * Non-respondents included 156 couples (34 births) who returned the refusal sheet.

Among the 6,507 couples who began an IVF program in 2000-2002, the contact rate was 62% (*n* = 4,029). Among contacted couples, the response rate was 58% (*n* = 2,321). Therefore, 36% of the initial cohort participated in the postal study (Figure 1). The proportion of couples who had a child during IVF treatment was higher among contacted (44%) than among non-contactable couples (38%) and it was higher among respondents (53%) than among non-respondents (31%).

Factors associated with survey contact are presented in Table 1. The proportion of contacted couples increased from 56% in younger women (< 30 years) to 64% in older women (≥ 40 years) and varied according to inclusion center from 52% to 72%. The more recent the first oocyte retrieval, the higher the proportion of contacted couples, ranging from 58% to 66%. In univariate as well as in multivariate analyses, all factors were associated with the probability of contact, except origin of infertility. In multivariate analysis, a live birth after IVF remained associated with probability of contact with an OR of 1.34 (95% CI [1.20; 1.50]).

**Table 1:** **Factors associated with probability of contact in the study (*n*** **= 6,507)**

		**Univariate analysis**			**Multivariate analysis (*n*** **= 6,231)**		
	**%**	**OR**	**95% CI**	***P***	**OR**	**95% CI**	***P***
**Woman’s age (years)**				<0.001*			<0.001*
< 30	56	1			1		
30-34	64	1.36	1.20-1.55		1.36	1.18-1.56	
35-39	64	1.38	1.20-1.59		1.51	1.30-1.75	
≥ 40	64	1.42	1.18-1.70		1.65	1.34-2.03	
**Inclusion center**				<0.001			<0.001
Marseille	63	1			1		
Bois-Guillaume	52	0.65	0.56-0.76		0.69	0.58-0.81	
Sèvres	61	0.94	0.79-1.11		0.99	0.82-1.20	
Besançon	55	0.72	0.60-0.87		0.84	0.69-1.02	
Caen	72	1.57	1.28-1.92		1.83	1.49-2.25	
Cochin	62	0.95	0.77-1.18		1.05	0.84-1.32	
Clermont-Ferrand	70	1.38	1.12-1.69		1.39	1.12-1.72	
Montsouris	69	1.31	1.07-1.60		1.32	1.08-1.63	
**Year of 1**st **oocyte retrieval**				<0.001*			<0.001*
2000	58	1			1		
2001	61	1.20	1.06-1.36		1.16	1.01-1.33	
2002	66	1.59	1.40-1.81		1.57	1.36-1.80	
**Origin of infertility**				0.22			0.82
Female	62	1			1		
Male	63	1.07	0.94-1.20		0.99	0.87-1.13	
Couple	62	1.00	0.86-1.16		0.98	0.84-1.14	
Unexplained	59	0.89	0.76-1.05		0.92	0.77-1.10	
**Number of embryos obtained at 1stattempt**				0.004*			<0.001*
0-1	59	1			1		
2-5	62	1.09	0.95-1.25		1.08	0.93-1.25	
> 5	64	1.22	1.06-1.41		1.29	1.11-1.51	
**Number of attempts**				<0.001*			<0.001*
1	56	1			1		
2-4	64	1.43	1.28-1.58		1.51	1.35-1.69	
>4	76	2.59	2.08-3.23		2.98	2.37-3.76	
**Result of IVF**							
No live birth	60	1			1		
≥ 1 live birth	65	1.27	1.15-1.41	<0.001	1.34	1.20-1.50	<0.001

* *P* for trend.

Factors associated with response to the postal survey are presented in Table 2. The proportion of respondents varied from 48% to 69% according to inclusion center. It increased with the total number of embryos transferred at first attempt and also appeared to be greater when the first oocyte retrieval was more recent. In multivariate analysis, a live birth following IVF was associated with the probability of response with an OR of 2.26 (95% CI [1.96; 2.60]).

**Table 2:** **Factors associated with probability of response to the postal questionnaire (*n*** **= 4,029)**

		**Univariate analysis**			**Multivariate analysis (*n*** **= 3,870)**		
	**%**	**OR**	**95% CI**	***P***	**OR**	**95% CI**	***P***
**Woman’s age (years)**				<0.001			0.001
< 30	60	1			1		
30-34	65	1.23	1.04-1.46		1.31	1.10-1.57	
35-39	53	0.77	0.64-0.92		0.95	0.79-1.16	
≥ 40	39	0.44	0.34-0.55		0.68	0.52-0.88	
**Inclusion center**				<0.001			<0.001
Marseille	53	1			1		
Bois-Guillaume	63	1.49	1.21-1.84		1.56	1.25-1.94	
Sèvres	54	1.03	0.83-1.27		1.18	0.92-1.51	
Besançon	64	1.55	1.20-2.00		1.53	1.17-2.02	
Caen	69	1.97	1.56-2.49		2.05	1.60-2.62	
Cochin	50	0.86	0.66-1.12		0.97	0.74-1.28	
Clermont-Ferrand	63	1.47	1.16-1.86		1.29	1.00-1.66	
Montsouris	48	0.79	0.63-0.99		0.85	0.67-1.08	
**Year of 1**st **oocyte retrieval**				0.03*			0.002*
2000	56	1			1		
2001	57	1.05	0.89-1.23		1.10	0.92-1.32	
2002	60	1.18	1.01-1.39		1.31	1.09-1.56	
**Origin of infertility**				0.75			0.14
Female	57	1			1		
Male	58	1.02	0.88-1.19		0.95	0.80-1.11	
Couple	57	1.01	0.84-1.21		0.93	0.76-1.13	
Unexplained	60	1.12	0.91-1.38		1.22	0.97-1.53	
**Number of embryos obtained at 1stattempt**				<0.001*			0.40*
0-1	55	1			1		
2-5	56	1.04	0.87-1.24		0.97	0.81-1.17	
> 5	62	1.34	1.12-1.61		1.06	0.87-1.30	
**Number of attempts**				0.61*			0.04*
1	57	1			1		
2-4	58	1.06	0.92-1.21		1.12	0.97-1.30	
>4	57	1.02	0.81-1.28		1.27	0.99-1.63	
**Result of IVF**							
No live birth	48	1			1		
≥ 1 live birth	70	2.44	2.14-2.78	<0.001	2.26	1.96-2.61	<0.001

* *P* for trend.

To check the stability of our results among unsuccessfully treated couples, multivariate analyses were conducted a second time, but only among unsuccessfully treated couples, and thus after having removed the variable “result of IVF”. Multivariate analyses of factors associated with contact (n = 3,597) and of factors associated with response (n = 2,274) among unsuccessfully treated couples are presented in Tables 3 and 4, respectively. Results regarding the different variables (other than result of IVF) among unsuccessfully treated couples are very close to those observed for the whole cohort for probability of contact as well as for probability of response.

**Table 3:** Factors associated with probability of contact in the study among unsuccessfully treated couples (n = 3,597)

	**Multivariate analysis**		
	**OR**	**95% CI**	***P***
**Woman’s age (years)**			<0.001*
< 30	1		
30-34	1.46	1.21-1.77	
35-39	1.50	1.23-1.83	
≥ 40	1.69	1.33-2.15	
**Inclusion center**			<0.001
Marseille	1		
Bois-Guillaume	0.79	0.63-0.97	
Sèvres	1.06	0.83-1.37	
Besançon	0.99	0.76-1.30	
Caen	1.83	1.40-2.41	
Cochin	1.07	0.82-1.39	
Clermont-Ferrand	1.18	0.89-1.56	
Montsouris	1.56	1.18-2.05	
**Year of 1**st **oocyte retrieval**			<0.001*
2000	1		
2001	1.13	0.94-1.35	
2002	1.43	1.19-1.72	
**Origin of infertility**			0.206
Female	1		
Male	1.03	0.97-1.23	
Couple	0.90	0.74-1.09	
Unexplained	0.83	0.67-1.04	
**Number of embryos obtained at 1stattempt**			0.009*
0-1	1		
2-5	1.10	0.93-1.31	
> 5	1.29	1.06-1.55	
**Number of attempts**			<0.001*
1	1		
2-4	1.59	1.37-1.84	
>4	3.76	2.78-5.08	

* P for trend.

**Table 4:** **Factors associated with probability of response to the postal questionnaire among unsuccessfully treated couples (*n*** **= 2,152)**

	**Multivariate analysis**		
	**OR**	**95% CI**	***P***
**Woman’s age (years)**			0.001
< 30	1		
30-34	1.48	1.15-1.90	
35-39	1.01	0.78-1.31	
≥ 40	0.64	0.47-0.88	
**Inclusion center**			<0.001
Marseille	1		
Bois-Guillaume	1.76	1.32-2.34	
Sèvres	1.37	0.99-1.88	
Besançon	1.33	0.93-1.91	
Caen	2.18	1.58-3.00	
Cochin	0.88	0.62-1.24	
Clermont-Ferrand	1.50	1.06-2.12	
Montsouris	0.96	0.70-1.33	
**Year of 1**st **oocyte retrieval**			0.003
2000	1		
2001	1.45	1.14-1.83	
2002	1.44	1.14-1.82	
**Origin of infertility**			0.14
Female	1		
Male	0.85	0.69-1.06	
Couple	0.80	0.62-1.03	
Unexplained	1.09	0.81-1.46	
**Number of embryos obtained at 1stattempt**			0.79
0-1	1		
2-5	1.06	0.85-1.32	
> 5	1.08	0.85-1.38	
**Number of attempts**			0.008*
1	1		
2-4	1.25	1.03-1.53	
>4	1.44	1.06-1.97	

* P for trend.

## Discussion

In the DAIFI cohort, a large retrospective cohort of 6,507 couples who began IVF treatment between 2000 and 2002, 36% of the initial cohort participated in a postal questionnaire survey 6 to 9 years later, after the first or second mailing; 38% of the cohort members could not be contacted and 26% were contacted but did not respond.

Thirty-eight percent of cohort members could not be contacted 6 to 9 years after beginning IVF treatment because they had moved to a new address. This proportion of lost to follow-up is similar to that observed in other studies of couples after IVF treatment. Among 1,614 eligible German couples, 44% could not be contacted 5 years after the birth of their ICSI child [25]. Among 475 eligible English couples, 25.5% could not be contacted 4 to 10 years after referral to a fertility clinic [26]. Obviously, the issue of loss to follow-up is not specific to IVF populations. For example, in the NEMESIS study investigating mental health in the general population in the Netherlands, 20% of attrition in the second wave was due to failure to locate or to contact respondents after only one year of follow-up [27]. To mitigate the problem of contact, most prospective cohorts use processes such as annual update of address or contact details of relatives or friends [28,29]. However, even with efforts to trace participants, in the Australian Longitudinal Study on Women’s Health 21% of 18- to 23-year-old women could not be contacted 4 years after the first survey [8]. It is thus important to understand factors associated with non-contact. We observed a roughly linear relation between the woman’s age and the probability of contact, corresponding to greater mobility of younger couples, a finding which is in agreement with previous studies [5,27]. As could logically be expected, women who had more recently begun IVF treatment in their inclusion center were more likely to be contacted (as they had shorter duration of follow-up), as were women with more numerous IVF attempts because they had left the center more recently and so also had a shorter duration of follow-up. The association between inclusion center and probability of contact may be linked to differences between centers in financial and human resources devoted to patient address update. It may also reflect the geographic location of the center as well as population dynamics, with mobility rates that can vary widely between regions. For instance, a change of address may be more likely in more urbanized areas [27]. Lack of association between origin of infertility and contact suggests that medical factors do not have an impact on contact. Nevertheless, the association that we observed between the total number of embryos obtained at first attempt and probability of contact was an unexpected finding, as was the association with having a child after IVF treatment. The greater probability of contact among couples who had a live birth following treatment is particularly surprising, because a birth is one of the reasons for a change of address (need for one more bedroom). A higher rate of relocation among couples who did not have a child during IVF treatment could partly be due to a higher rate of couple separation. Such a hypothesis would need to be confirmed by further research.

Among the 4,029 couples contacted, 58% responded to the postal questionnaire. This rate appears similar to the few reported response rates among contacted couples in studies of IVF couples, and which ranged from 44% to 75% [23,26,30,31]. In an IVF population, non-response could be linked to the physical and psychological burden of IVF treatment, especially when the treatment has not led to the expected live birth [23]. However, some similar response rates have been reported in studies among young women. For example, in the Australian Longitudinal Study on Woman’s Health, 64% of women aged 18 to 23 years responded seven years after the first survey [17]. Another recent study among uninsured women aged 15 to 44 years reported a response rate of 61% with a median follow-up of only 2.4 years [32]. These results in fact led us to question the hypothesis that a lower response rate among IVF couples may be linked to the burden of treatment. Regarding factors associated with response, in our study an inverse-J relation was observed between the woman’s age and the probability of response. A similar relationship has been demonstrated between the woman’s age and the IVF live-birth rate [33,34]. The inverse J-pattern between age and response suggests that age impacts as a medical factor on probability of response. Probability of response was also associated with inclusion center. Differences observed between centers may reflect in part couples’ feelings on their IVF treatment in the center, but probably also reflect sociodemographic characteristics of couples that may vary according to geographic location. Indeed, socioeconomic and educational levels are known to be associated with response rates in epidemiological studies [1,8]. The trend toward a higher response rate among couples with unexplained infertility than in couples with infertility of female origin also suggests that demographic and medical factors influence contact and response in different ways. Our results may appear to differ from those of Cahill et al., who found that response rate to a postal questionnaire 4 to 10 years after referral to an IVF center was not significantly affected by the woman’s age, duration of infertility or ever having been pregnant or not [26]. However, in this English study, lack of significant differences may be due to a lack of power, as the analyses were conducted on a small sample (*n* = 354). Participation was found to be strongly associated with birth of a child during treatment, indicating that there was a selection bias among the respondents to the postal survey. When the frequency of parenthood project achievement is being estimated, methods such as multiple imputation, that can adjusted for non-participation, should be used.

## Conclusion

It is necessary to understand the mechanisms underlying contact and response in order to choose the appropriate methodology for analysis of the results of epidemiological surveys [35]. To take into account attrition and potential bias, new methods are being developed but most rely on hypotheses that require an understanding of attrition mechanisms [36]. Studies on attrition mechanisms are needed, especially as these mechanisms may vary according to the study population. In our study based on infertile couples treated by IVF, we found that an a priori hypothesis on attrition may be too simplistic and may underestimate potential bias. In our study, non-response as well as non-contact were linked to the outcome of interest. Attrition is a common issue in all health surveys and one that is rarely addressed in analysis. This study illustrates the importance of developing a study design that yields a minimum of information on the whole of the eligible population. In the context of growing use of analytical methods that take attrition into account (such as multiple imputation), we need to better understand the mechanisms that underlie attrition in order to choose the most appropriate method.

## Abbreviations

ICSI: Intracytoplasmic sperm injection; IVF: In vitro fertilization; OR: Odds-ratio.

## Competing interests

None of the authors have any conflict of interest.

This study was not sponsored or funded by industry. It received funding only from French government agencies.

## Authors’ contributions

P. Troude designed the study’s analytic strategy, conducted literature review, conducted the statistical analysis, and drafted the manuscript. E. Bailly participated in data collection, reviewed the study’s analytic strategy and reviewed the manuscript. J. Guibert coordinated the study in fertility centers, reviewed the study’s analytic strategy and results, and reviewed the manuscript. J. Bouyer designed the study’s analytic strategy, reviewed results, and reviewed the manuscript. E. de La Rochebrochard designed the study, designed the study’s analytic strategy, and drafted the manuscript. She had full access to all of the data in the study and takes responsibility for the integrity of the data and the accuracy of the data analysis All members of the DAIFI group reviewed the manuscript.

## Authors’ information

DAIFI Group members include: Institut National d’Etudes Démographiques (INED) - Institut National de la Santé et de la Recherche Médicale (INSERM) – Université Paris Sud XI: Elise de La Rochebrochard (national coordinator), Estelle Bailly, Jean Bouyer, Juliette Guibert, Henri Leridon, Patricia Thauvin, Laurent Toulemon, Pénélope Troude; Auvergne: Rusudan Peikrishvili, Jean-Luc Pouly (CHU Estaing, Clermont-Ferrand 63); Basse-Normandie: Isabelle Denis, Michel Herlicoviez (CHU Clémenceau, Caen 14); Franche-Comté: Christiane Joanne, Christophe Roux (CHR Saint-Jacques, Besançon 25); Haute-Normandie: Catherine Avril, Julie Roset (Clinique Saint-Antoine, Bois-Guillaume 76); Ile-de-France: Joëlle Belaisch-Allart, Olivier Kulski (Centre Hospitalier des 4 Villes, Sèvres 92); Jean-Philippe Wolf, Dominique de Ziegler (Cochin, Paris 75); Philippe Granet, Juliette Guibert (Institut Mutualiste Montsouris, Paris 75); Provence-Alpes-Côte d’Azur: Claude Giorgetti, Géraldine Porcu (Institut de Médecine de la Reproduction, IMR, Marseille 13).

## Pre-publication history

The pre-publication history for this paper can be accessed here:

http://www.biomedcentral.com/1471-2288/12/104/prepub
